# Low power laser irradiation and human adipose-derived stem cell treatments promote bone regeneration in critical-sized calvarial defects in rats

**DOI:** 10.1371/journal.pone.0195337

**Published:** 2018-04-05

**Authors:** Yan-Hsiung Wang, Jyun-Yi Wu, Su Chii Kong, Min-Hsuan Chiang, Mei-Ling Ho, Ming-Long Yeh, Chia-Hsin Chen

**Affiliations:** 1 School of Dentistry, College of Dental Medicine, Kaohsiung Medical University, Kaohsiung, Taiwan; 2 Orthopaedic Research Center, College of Medicine, Kaohsiung Medical University, Kaohsiung, Taiwan; 3 Department of Medical Research, Kaohsiung Medical University Hospital, Kaohsiung Medical University, Kaohsiung, Taiwan; 4 Fresenius Kabi Taiwan Ltd, Taipei, Taiwan; 5 Department of Physiology, College of Medicine, Kaohsiung Medical University, Kaohsiung, Taiwan; 6 Department of Marine Biotechnology and Resources, National Sun Yat-sen University, Kaohsiung, Taiwan; 7 Department of Biomedical Engineering, National Cheng Kung University, Tainan, Taiwan; 8 Medical Device Innovation Center, National Cheng Kung University, Tainan, Taiwan; 9 Department of Physical Medicine and Rehabilitation, School of Medicine, College of Medicine, Kaohsiung Medical University, Kaohsiung, Taiwan; 10 Department of Physical Medicine and Rehabilitation, Kaohsiung Medical University Hospital, Kaohsiung Medical University, Kaohsiung, Taiwan; Università degli Studi della Campania, ITALY

## Abstract

Both stem cell therapy and physical treatments have been shown to be beneficial in accelerating bone healing. However, the efficacy of combined treatment with stem cells and physical stimuli for large bone defects remains uncertain. The aim of this study was to evaluate the bone regeneration effects of low-power laser irradiation (LPLI) and human adipose-derived stem cell (ADSC) treatments during fracture repair using a comparative rat calvarial defect model. We evaluated the viability of human ADSCs, which were cultured on a porous PLGA scaffold using an MTS assay. The critical-sized calvarial bone defect rats were divided into 4 groups: control group, LPLI group, ADSC group, and ADSC+LPLI group. Bone formation was evaluated using micro-CT. New bone formation areas and osteogenic factor expression levels were then examined by histomorphological analysis and immunohistochemical staining. Our data showed that PLGA had no cytotoxic effect on human ADSCs. Micro-CT analyses revealed that both the LPLI and ADSC groups showed improved calvarial bone defect healing compared to the control group. In addition, the ADSC+LPLI group showed significantly increased bone volume at 16 weeks after surgery. The area of new bone formation ranked as follows: control group < LPLI group < ADSC group < ADSC+LPLI group. There were significant differences between the groups. In addition, both ADSC and ADSC+LPLI groups showed strong signals of vWF expression. ADSC and LPLI treatments improved fracture repair in critical-sized calvarial defects in rats. Importantly, the combined treatment of ADSCs and LPLI further enhances the bone healing process.

## Introduction

Critical-sized bone defects are formed when the bone loses its intrinsic capacity to spontaneously heal, leading to extensive bone loss; once such defects occur, external interventions are necessary for healing to progress [1]. These large bone defects often arise following trauma, non-union, debridement of infections, resection of tumors or periprosthetic osteolysis [2]. The resources of bone grafts, which are commonly used in the treatment of critical-sized bone defects, can be generated by autologous, homologous, and heterologous methods. However, these resources are often accompanied by various limitations and challenging post-operative complications, such as disease transmission and immunologic reactions. Consequently, these limitations have promoted a continuous search for bone graft substitutes and alternative therapies directed at the repair and regeneration of bone.

Mesenchymal stem cell (MSC) therapy is being actively investigated for bone fracture repair due to the known abilities of MSCs in osteogenic and chondrogenic differentiation during bone formation[3, 4]. Adipose tissue-derived stem cells (ADSCs) are stromal cell populations isolated from adipose tissues that are morphologically and phenotypically similar to the MSCs [5]. ADSCs exhibit a high potential ability for differentiation into multilineage cells, such as osteoblasts, chondrocytes, myocytes and adipocytes [6–8]. The extraction of ADSCs is relatively simple, with minimum donor site morbidity. More importantly, compared to other age-dependent adult stem cells, such as bone marrow-derived mesenchymal stem cells (BMSCs), the quality and proliferation capacities of ADSCs do not decline with the age of the patient [9–11]. ADSCs are therefore a superior cell to other types of adult stem cells as a source for clinical approaches.

Low power laser irradiation (LPLI) consists of non-thermal irradiation at wavelengths between the visible and near-infrared spectra [12, 13]. The clinical application of LPLI adopts the principle of photobiomodulation to improve tissue healing and regeneration. The biostimulating effects of this technique are achieved when a specific wavelength of light is absorbed by the photoreceptors of the irradiated cells [14]. A wide network of intracellular signaling cascades are then triggered, leading to various biological effects in promoting cell growth and survival, proliferation, collagen synthesis, and differentiation [15–18]. Studies have reported that LPLI exerts biostimulating effects on osteogenic cells and bone tissues *in vitro* [15, 19–22]. Other studies have also demonstrated that LPLI affects osteogenesis and bone repair in comparative animal models [23–28]. LPLI therapies also induce alkaline phosphatase activity and increase osteocalcin gene expression, thus contributing to bone formation [29–31].

The aim of the current study was to evaluate the effects of LPLI and human ADSCs in bone regeneration during fracture repair using a comparative rat calvarial defect model of fracture healing. Several previous studies have employed the use of MSCs in combination with an osteoconductive 3D scaffold to examine the bone regenerative capacity [32, 33]. Here, we applied porous biodegradable poly-lactic-co-glycolic acid (PLGA) as a scaffold for culturing ADSCs in osteogenic media, which were later implanted in the created defect sites on the rat calvaria.

## Materials and methods

### Laser apparatus

**Fig 1:**
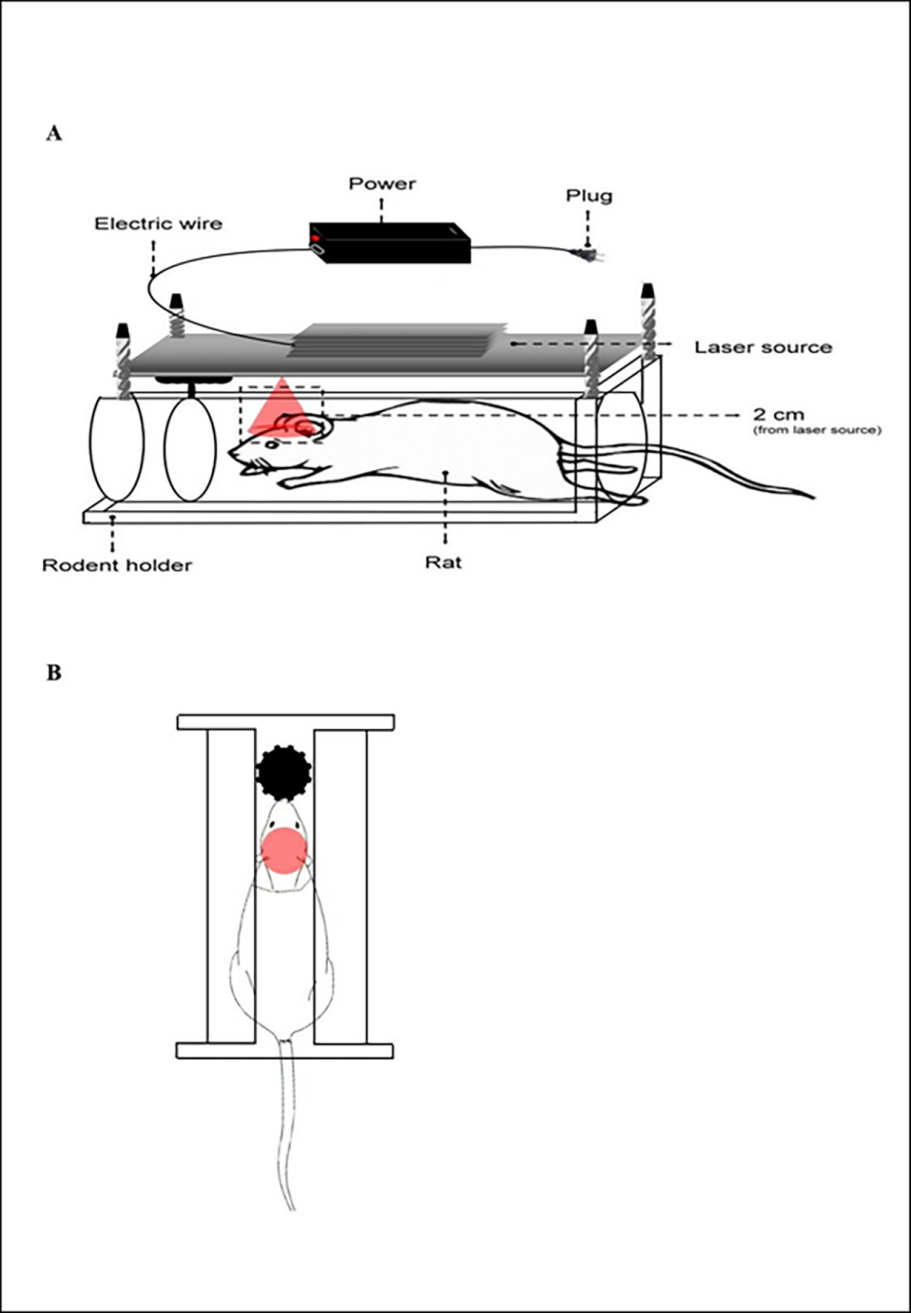
Schematic diagram of the laser apparatus. **(A)** Side view and **(B)** top view of the laser apparatus applied in the *in vivo* experiments. Rats were immobilized in the mouse holder. Pillars were adjusted to cover the target defect area of irradiation.

A gallium-aluminum-arsenide (GaAlAs) red laser (wavelength 660 nm) (TRANSVERSE IND. CO., LTD., Taipei, Taiwan) was used as a light source, as detailed in a previous study [12]. The laser device was designed to simultaneously produce 12 laser beams with a power of 70 mW for use in cell experiments, as described in previous studies [12, 34, 35]. In the *in vivo* animal experiments, the distance between the laser source and vertex of the rat was 2 cm. Under these conditions, the spot size of the laser was 2.64 cm^2^, which allowed the defect area of the calvarial bone (0.38465 cm^2^) to be fully covered (Fig 1). The power density was 24.62 mW/cm^2^, and the target site in rats was irradiated for 540 seconds to receive a final energy dose of 13.3 J/cm^2^. A higher energy dose exposure was applied as the laser beam diminished in energy fluency when penetrating the skin tissue in the *in vivo* treatment. The decay rate was approximately 70% in skin tissue based on a pilot study collected from a low-power laser-penetrating test (data not shown). Consequently, when the 70% decay rate of the energies was deducted from the received energies of 13.3 J/cm^2^ (13.3 J/cm^2^ × 30% = 3.99 J/cm^2^), the final energy dose to actually irradiate the bone defect site of the calvaria was 4 J/cm^2^.

### Human adipose-derived stem cell culture

Human adipose-derived stem cells (hADSCs) were purchased from Cellular Engineering Technologies (CET, Coralville, IA, USA). The hADSCs were cultured and expanded in a BK medium containing keratinocyte serum-free medium (KSFM, GIBCO-BRL, Rockville, MD, USA) supplemented with bovine pituitary extract (25 mg), human recombinant EGF protein (2.5 μg), 0.2 mM N-acetyl-Cysteine (NAC, Sigma-Aldrich, Saint Louis, MO, USA), 0.2 mM L-Ascorbic acid 2-phosphate magnesium (LA2-P, Sigma-Aldrich, Saint Louis, MO, USA), 5% FBS (SAFC Biosciences, Saint Louis, MO, USA), and 1% penicillin/streptomycin (GIBCO-BRL, Rockville, MD, USA). The medium was further mixed at a ratio of 1:1 with a mixture of DMEM with low glucose (GIBCO-BRL, Rockville, MD, USA) containing 0.06 μg/mL insulin (Sigma-Aldrich, Saint Louis, MO, USA), 5% FBS, and 1% penicillin/streptomycin. The cells were cultured in a humidified 5% CO_2_ atmosphere in a 37°C incubator, and the medium was changed every other day. Cells within ten passages were used in the experiments.

### Fabrication of PLGA scaffolds

**Fig 2:**
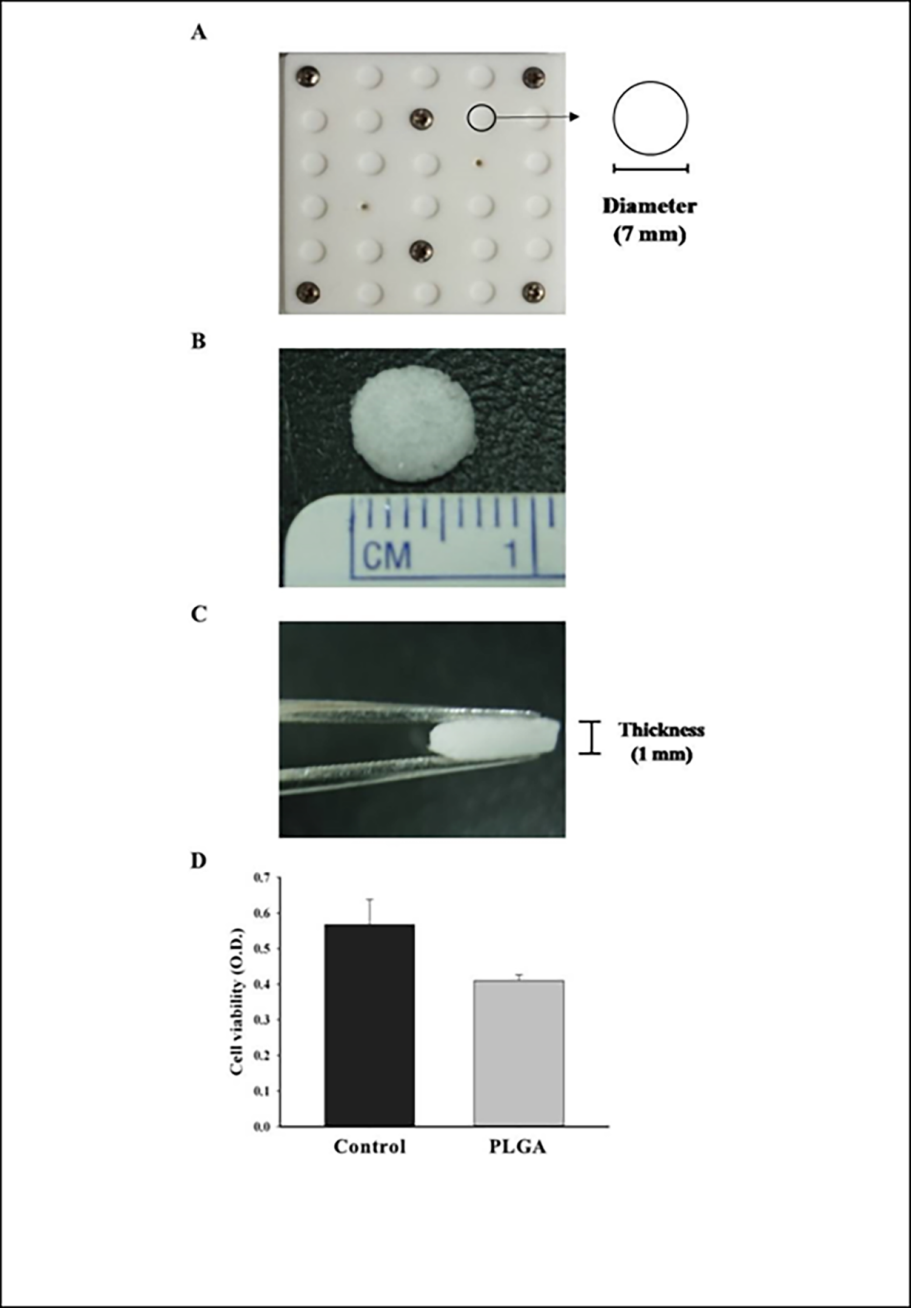
Fabrication of the PLGA scaffolds. **(A)** The disc-shaped Teflon cylinder molds used for the fabrication of porous PLGA scaffolds. **(B)** The top view and diameter (7 mm) of the fabricated PLGA scaffolds. **(C)** The side view and thickness (1 mm) of the fabricated PLGA scaffolds. **(D)** The hADSCs were seeded onto culture plates (Control) or PLGA scaffolds (PLGA). After 24 hours of culture, the MTS assay was performed to evaluate the cell viability (n = 3).

Porous PLGA scaffolds (with a lactide/glycolide ratio of 85/15 and molecular weight of 50–75 kDa, Sigma, St. Louis, MO, USA) were fabricated in accordance with a previously described technique [36], with some modifications. Briefly, 0.4 g PLGA powder was dissolved in 2 mL of chloroform (J.T. Backer, Phillipsburg, NJ, USA), and 3.6 g 90% of NaCl salts (particle size was smaller than 300 μm) were added under magnetic stirring to form a 20% w/v PLGA solution. Then, the mixture was poured into disc-shaped Teflon cylinder molds (Fig 2A) and lyophilized at -20°C for one day (or -60°C overnight) to remove the chloroform and form the PLGA sponges. The molds were then immersed in deionized water to induce salt leaching within the polymer/salt matrices. Finally, the porous PLGA scaffolds (1 mm in thickness and 7 mm in diameter) (Fig 2B and 2C) were removed from the molds and washed with distilled water at least two times for 48 hours. After drying under vacuum conditions, the porous PLGA scaffolds were stored at -20°C.

### Cell culture on the PLGA scaffolds

The PLGA scaffolds were pre-wetted and sterilized with an aqueous solution of 75% (v/v) ethanol and subsequently plated onto 3.5-cm dishes. Cell suspensions at a density of 6 × 10^6^ cells per 1 mL were pipetted onto the top surface of each pre-wetted scaffold and allowed to penetrate into the scaffold. The cell/scaffold constructs were then incubated in a BK medium at 37°C under 5% CO_2_ overnight (12 hours) to ensure cell adherence. After cell adherence, the cell/scaffold constructs were placed into the calvarial defects generated as described below.

### MTS assay

MTS is a colorimetric method for determining the number of viable cells. In this study, the CellTiter 96® AQueous One Solution Cell Proliferation Assay (Promega, Madison, WI, USA) containing MTS compound and an electron coupling reagent phenazine ethosulfate (PES) was used. 20 μL of MTS solution per 100 μL of culture medium was directly added to each well of the assay plate. Then, the plate was incubated for 3 hours in a humidified 5% CO_2_ atmosphere in a 37°C incubator. Finally, the absorbance values were measured using an ELISA reader (Model no. 680, Bio-Rad) at 490 nm.

### Animals

Sprague-Dawley (SD) rats were used in this study for the *in vivo* animal experiments. The rats were purchased from the National Laboratory Center (Taipei, Taiwan), and the experiments were approved by the Kaohsiung Medical University Animal Care and Use Committee (affidavit of approval of animal use protocol number: 100054). The rats were bred in a stainless steel cage of 35 cm (length) × 25 cm (width) × 22 cm (height) and maintained in the animal room with a relative humidity of 65 ± 5% and temperature of 25 ± 2°C. The rats were maintained under in a constant light and dark cycle, with the light period from 06:00 a.m. to 18:00 p.m. and dark period from 18:00 p.m. to 06:00 a.m. the following day.

### Animal procedures

Twenty-four eight-week-old male SD rats were used to study the effects of LPLI on bone regeneration for repairing calvarial defects. Cell/scaffold constructs were implanted under anesthesia with an intra-peritoneal injection of ketamine (Ketalar Parke-Davis, Taiwan) in combination with xylazine hydrochloride (Rompun Bayer HealthCare, Germany) at a ratio of 1:1 (1 μL/g body weight). Before the operation, the hair over the calvarium was removed by using an electric shaver, and the area was cleaned with 70% ethanol. A midline calvarial incision was made, and a 7-mm hole was drilled to penetrate the calvarial bone using a trephine burr with constantly iced PBS irrigation. Extreme care was taken to avoid damaging the dura mater. Twenty-four rats with calvarial defects were randomly designated into 4 groups (6 rats/group) based on the following cell/scaffold implants and LPLI treatments: the Control group, the PLGA scaffold alone (without LPLI treatment) group; the LPLI group, the PLGA scaffold+LPLI (4 J/cm^2^) group; the ADSC group, the PLGA +ADSCs group; and the ADSC+LPLI group, PLGA scaffold+ADSCs+LPLI (4 J/cm^2^).

The dura mater and skin wounds were sutured after implantation with nylon 5–0 and 4–0 sutures. At 24 hours post-implantation, the rats were immobilized in the mouse holder and LPLI at an energy dose of 4 J/cm^2^ was locally irradiated to the centers of the calvarial defects daily for 16 weeks. All rats survived without wound infections throughout the experiment. Sixteen weeks after implantation, all rats were euthanized using CO_2_ inhalation. Calvarial specimens were harvested and fixed in 10% neutral buffered formalin (TONYAR BIOTECH. INC., Taoyuan, Taiwan) at 4°C for 48 hours.

### Micro-CT analysis

Both qualitative and quantitative measurements of the bone regeneration level within the calvarial defects were obtained by a high-resolution microtomograph 1076 scanner (Skyscan, Kontich, Belgium). All rats in the 4 groups were analyzed at 0, 4, 8, 12, and 16 weeks. The living rats were anesthetized by intra-peritoneal injection of ketamine/ xylazine hydrochloride (1:1, 1 μL/g body weight) and fixed on a sample holder with the sagittal axis of the cranium perpendicular to the scanning plane. The voltage and beam currents of the X-ray source were set at 50 kV and 200 μA, respectively, as these parameters provided the best visualization. The samples were scanned through a 360° rotation angle at a pixel size of 35 μm. Skycan software, including NRecon version 1.6.4.0, Skyscan CT-Analyzer program version 1.11.4.2, CT Vol: Realistic 3D-Visualization version 1.11.1.2 and Data Viewer version 1.4.1, was used to reconstruct the image data, visualize the representation scan images, and quantify the newly formed bone. A cylindrical region of interest (ROI) measuring 9 mm in diameter and concentric to the defect site was selected for analysis according to the CT data set. This ROI covered the original defect region and the surrounding calvarial bone region. The volume of the bone growth was measured as the bone volume per total tissue volume (BV/TV).

### Histomorphological analysis

All rats were sacrificed 16 weeks after implantation, and the harvested calvaria were immediately fixed in 10% neutral-buffered formalin (TONYAR BIOTECH. INC., Taoyuan, Taiwan). After the last scan for micro-CT analysis, calvaria were subsequently decalcified with a 0.5 M EDTA in double-distilled water for 2 weeks. The samples were then dehydrated with a graded ethanol series (75%, 85%, 90%, 95%, and 100%) and paraffin-embedded. The resulting 5-μm thick sections were prepared and stained with hematoxylin and eosin (H&E, Sigma-Aldrich, Saint Louis, MO, USA). The stained sections were observed using light microscopy at 40X and 100X magnification, and the images were captured with a digital camera (Nikon, Tokyo, Japan). Finally, the percentage of the new bone formation area was measured using Image-Pro Plus 5.0 (Media Cybernetics, Silver Spring, MD, USA).

### Immunohistochemistry (IHC) analysis

IHC staining of endothelial vessels for von Willebrand factor (vWF) and osteogenic factors BMP-2 was then performed. Briefly, the sections were treated with 1 mg/mL pronase (Sigma-Aldrich, Missouri, USA) for 30 min and then incubated overnight at 4°C with a polyclonal rabbit anti-vWF (dilution 1:700; Millipore, MA, USA), anti-BMP-2 **(**dilution 1:700; Bioss Antibodies, MA, USA), anti-collagen I (dilution 1:100; Novus Biologicals, CO, USA) and anti-osteocalcin (dilution 1:100; Santa Cruz Biotechnology, CA, USA) antibodies, respectively. Secondary antibody goat anti-rabbit biotinylated immunoglobulin (dilution 1:1000; DakoCytomation, Denmark) was then applied for 60 min at 37°C. Streptavidin peroxidase (Vector Laboratories, Burlingame, US) was applied at a 1:1000 dilution for 60 min at 37°C. The peroxidase activity was detected using 0.4 mg/L of 3.3'-diaminobenzidine in a phosphate buffer, pH of 7.3, in the presence of 0.12% H_2_O_2_. The immunostaining signals were also measured using Image-Pro Plus 5.0 software.

### Statistical analysis

SPSS version 17.0 was used for statistical analysis. The results were expressed as the means ± standard error. Statistically significant differences were determined using the analysis of variance (ANOVA) followed by a *post hoc* Tukey’s test for multiple comparisons, and a *p*-value < 0.05 was considered statistically significant.

## Results

### Human ADSCs survived in the PLGA scaffold

We seeded 1.2 × 10^6^ hADSCs onto the PLGA scaffold in 48-well plates before implantation into the calvarial defect site. After culturing for 24 hours for cell adherence to form the cell/scaffold constructs, the cell viability was assessed using an MTS assay. The same cell number of ADSCs was also seeded onto culture plates without the scaffold as a control group. The results showed that most ADSCs were viable in the scaffold compared to the control group, indicating that seeding ADSCs onto the scaffold did not affect their viability; hence, PLGA is likely not toxic to the cells (Fig 2D).

### ADSC and/or LPLI treatments promote bone formation by micro-CT analysis

The effects of ADSC and LPLI treatments on bone repair were evaluated at 4, 8, 12, and 16 weeks post-operation using micro-CT analysis. Two parameters (bone volume and defect area) were analyzed by two range of interest (ROI) tests. To measure the bone volume, the ROI was defined to fit the ambit of the original calvarial defect (7 mm in diameter) at week 0 (S1 Fig, upper row). The same ROI was then used at other time points to measure the bone growth volume. A defect area of 9 mm (S1 Fig, lower row) was evaluated because the defect range was observed to have expanded as the animals continued to grow during the experimental process. Measuring the reduction in the defect area would enable further estimates of the bone repair rate. For this evaluation, the ROI ambit was enlarged to cover the defect site and surrounding calvarial bone (9 mm in diameter; S1 Fig, lower row).

**Fig 3:**
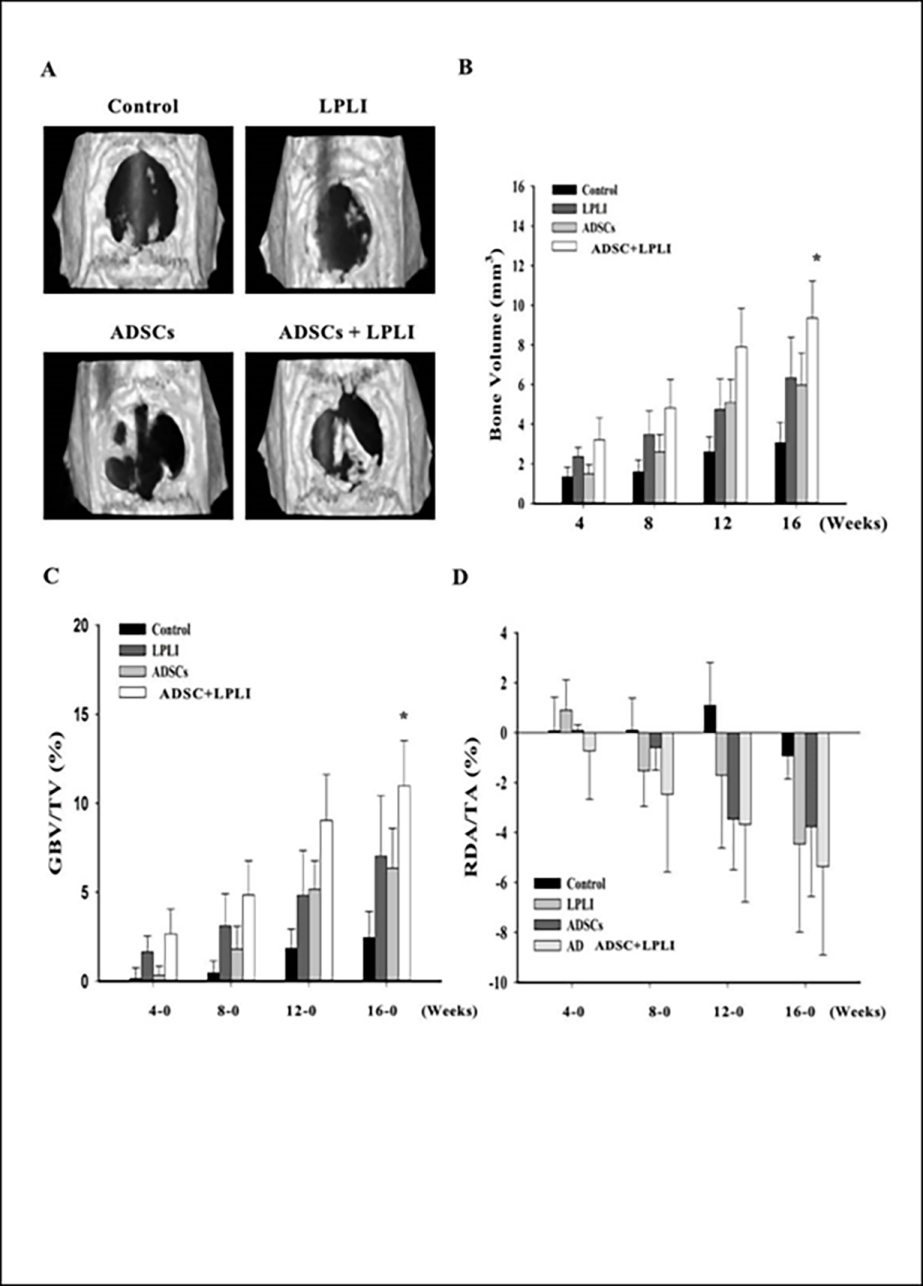
Analysis of bone repair metabolism in a rat calvarial bone defect model at 4, 8, 12, and 16 weeks post-operation. **(A)** Three-dimensional reconstructed micro-CT of the calvarial bone defects at 16 weeks post-operation. Representative photographs of the control, LPLI, ADSC, and ADSC+LPLI groups. **(B)** The volume of newly regenerated bone at each time point. The data are shown as the means ± SE (n = 6). **(C)** Morphometric analysis (GBV/TV) of new bone formation, which was normalized to BV at week 0. The data are shown as the means ± SE (n = 6). **(D)** Analysis of the relative ratio of the reduced defect area to the total ROI ambit (RDA/TA) area at each time point, which was normalized to the defect area at week 0. The data are shown as the means ± SE (n = 6). The following statistical level was applied: *p<0.05 compared to the control group.

Representative photographs of the 3D reconstructed micro-CT scans are shown in Fig 3A. To quantitate the extent of the regenerated bone, the bone volume for each group at each time point was measured, and quantitative data were collected as shown in Table 1. At week 0, the basal bone volume was counted as the selected ROI scope covering the edge of the surrounding calvarial bone. The bone volume from week 4 to 16 was shown to gradually increase with time in all experimental groups (Fig 3B). Quantitatively, the LPLI treatment group expressed similar bone formative ability as the ADSC group (Fig 3B). The LPLI group displayed more bone regeneration than either the non-ADSC-loaded groups (control *vs*. LPLI) or the ADSC-loaded groups (ADSCs *vs*. ADSCs+LPLI) (Fig 3B). The ADSC+LPLI group demonstrated the highest bone regeneration among all groups at all time points assessed. However, a significant difference in the measured bone volume between the control group and the ADSC+LPLI group was observed after 16 weeks.

**Table 1:** Newly regenerated bone volume assessed by micro-CT.

Weeks	0	4	8	12	16
Control	1.2296 ±0.2701	1.3481 ±0.5913	1.5928 ±0.7421	2.6094 ±0.9217	3.069 ±1.2557
LPLI	1.1672 ±0.2276	2.3688 ±0.5568	3.4831 ±1.4649	4.7451 ±1.8949	6.3472 ±2.4974
ADSC	1.2599 ±0.2092	1.5033 ±0.4582	2.599 ±0.8721	5.0952 ±1.1607	5.9772 ±1.6007
ADSC+LPLI	1.2345 ±0.1729	3.2108 ±1.1169	4.817 ±1.4455	7.9108 ±1.9373	9.3586 ±1.869

Bone volume, means ±SD mm^3^.

Next, the bone volume at each time point was normalized to that at week 0, which was defined as growth bone volume (GBV). The GBV relative to the total volume (GBV/TV) was also measured. The GBV/TV of the ADSC group was expressed to a similar degree compared to the control at week 4, but continuous increments were observed at 8, 12, and 16 weeks (Fig 3C). The results of GBV/TV expressed similar patterns to the bone volume. Only the ADSC+LPLI group at week 16 displayed a significantly higher amount of newly generated bone than the control group.

The defect area at each time point was normalized to that at week 0, which was defined as a reduced defect area (RDA). Then, the RDA relative to the total ROI area (RDA/TA) was measured. A RDA/TA ratio larger than zero indicates that the RDA continued to enlarge; in contrast, a value smaller than zero indicates that the size of the RDA was reduced. At week 4, only the ADSC+LPLI group showed a decreased defect area (Fig 3D). The size of the defect areas in the LPLI, ADSC, and ADSC+LPLI groups continued to decrease after 8 weeks, indicating that the bone formation process continued. However, the defect area of the control group remained until 12 weeks without any detectable healing (Fig 3D). The pattern of RDA/TA data was similar to that of the GBV/TV data shown in Fig 3C. The ADSC+LPLI group displayed better bone healing outcomes than the control group and the other experimental groups, but no significant difference was found.

### ADSC and/or LPLI treatments promote bone regeneration as shown by H&E analysis

**Fig 4:**
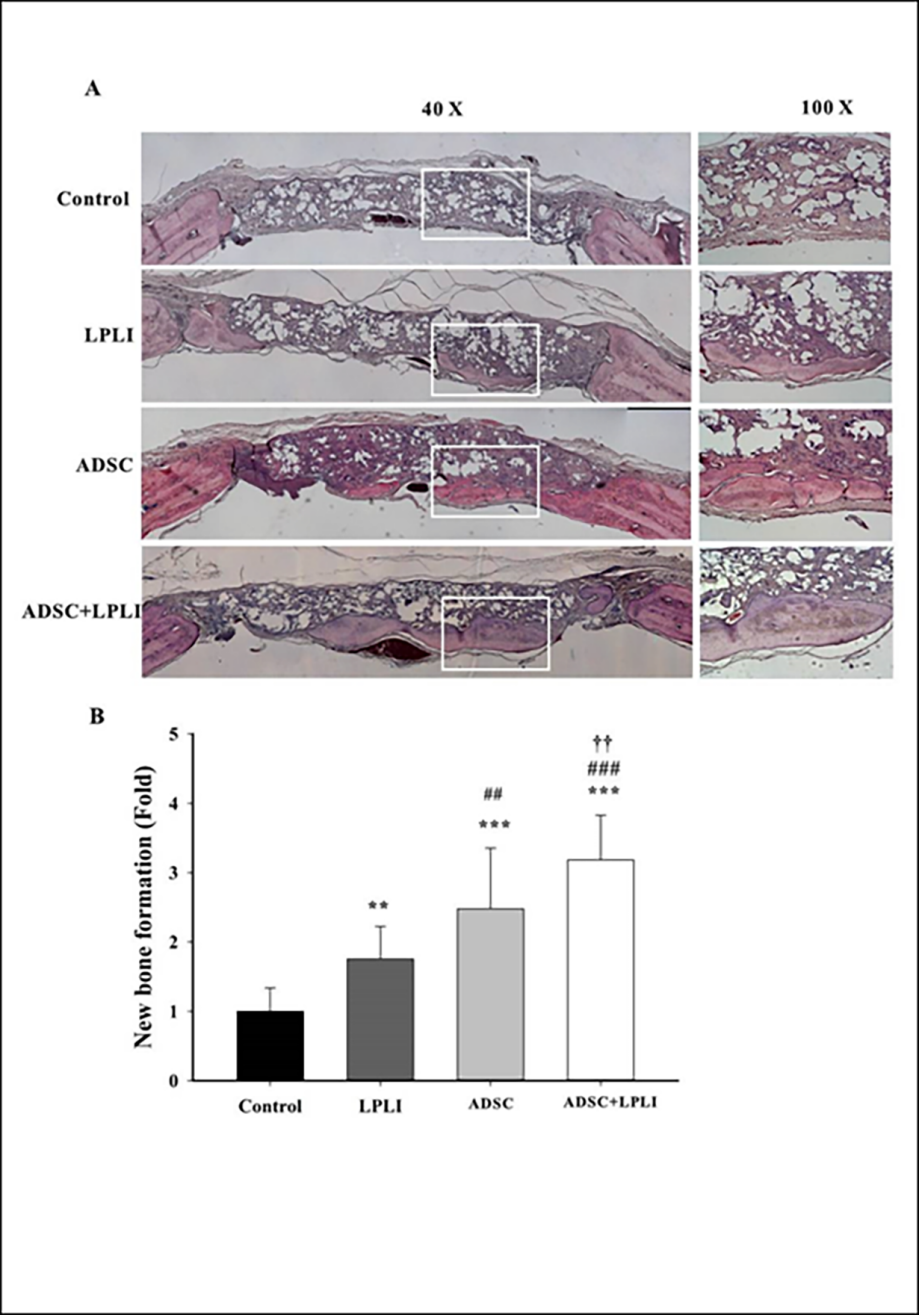
Histomorphological analysis of the calvarial defects at 16 weeks post-operation. **(A)** Representative H&E staining images of calvarial bone sections from each group: Control, LPLI, ADSC, and ADSC+LPLI treatment. The calvarial bone sections were collected at post-operative week 16 and stained using H&E. Right panel, representative sections with higher magnification from each group. *Bar*, 500 μm. **(B)** Quantitative measurements obtained from the H&E-stained images. The new bone formation area in the calvarial defect site was measured, and the percentage of bone matrix within the callus was calculated. Data were expressed as the means ± SE (n = 6), ***P<0.001.

The quality of regenerated bone at the calvarial defect sites was analyzed by H&E staining at 16 weeks post-operation. Representative images of the H&E-stained cross section of the calvarial defects are shown (Fig 4A). After 16 weeks, the defects in the control group were filled with thin, loose connective tissue (Fig 4A). Confirming the micro-CT observations, all treatments resulted in a significant increase in new bone formation originating from the defect margins at week 16. The best treatment for new bone formation was found in the ADSC+LPLI group. The new bone tissue was found inside and outside the scaffold. After image quantitation, a larger new bone area was observed in both the LPLI group and ADSC group compared to that in the control group, with the ADSC group showing higher bone formative ability than the LPLI group (Fig 4B). Moreover, the ADSC+LPLI group demonstrated the highest bone formative ability among the groups (Fig 4B). The area of new bone formation was displayed in the order of control group < LPLI group < ADSC group < ADSC+LPLI group, and a significant difference between the groups was observed.

### Immunohistomorphological analysis of blood vessel marker vWF and osteogenic factors

**Fig 5:**
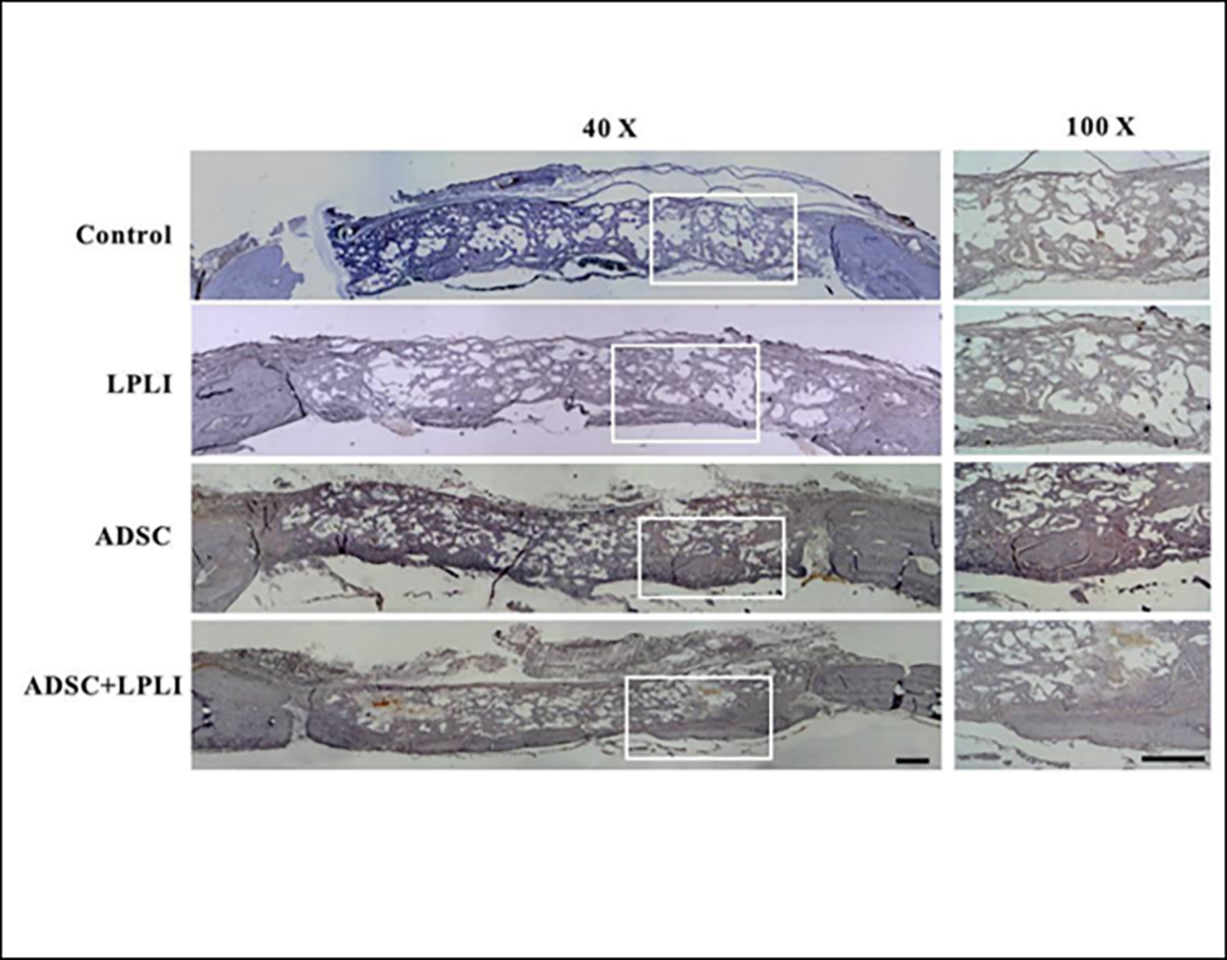
IHC staining of blood vessel marker vWF of the calvarial defects at 16 weeks post-operation. The calvarial bone sections were stained for vWF. Right panel, representative sections with a higher magnification in each group. *Bar*, 500 μm.

**Fig 6:**
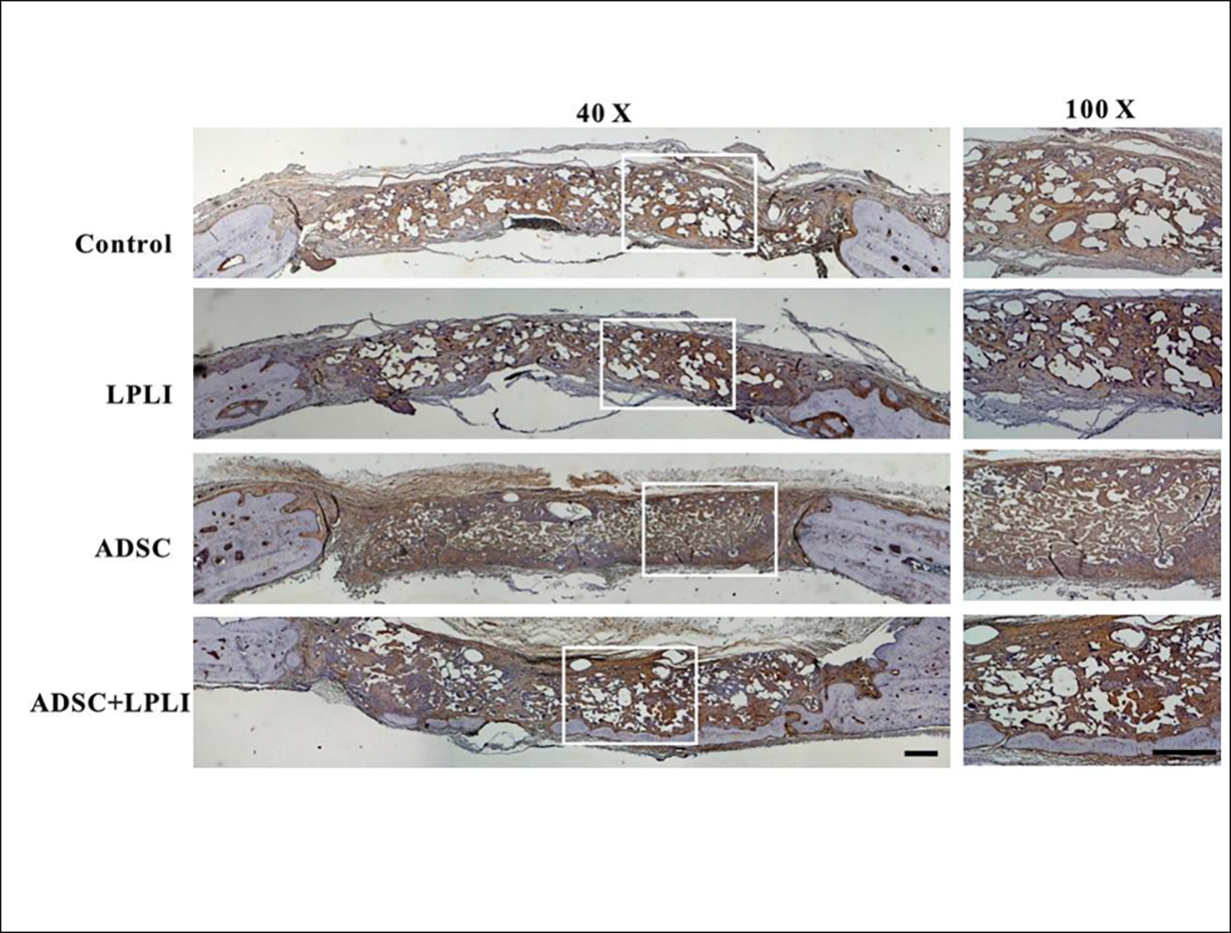
IHC staining of osteogenic factor BMP-2 of the calvarial defects at 16 weeks post-operation. The calvarial bone sections were stained for BMP-2. Right panel, representative sections with a higher magnification in each group. *Bar*, 500 μm.

**Fig 7:**
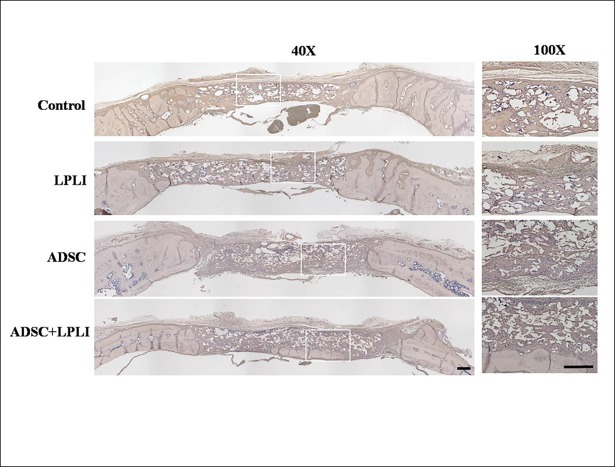
IHC staining of osteogenic factor collagen I of the calvarial defects at 16 weeks post-operation. The calvarial bone sections were stained for collagen I. Right panel, representative sections with a higher magnification in each group. *Bar*, 500 μm.

**Fig 8:**
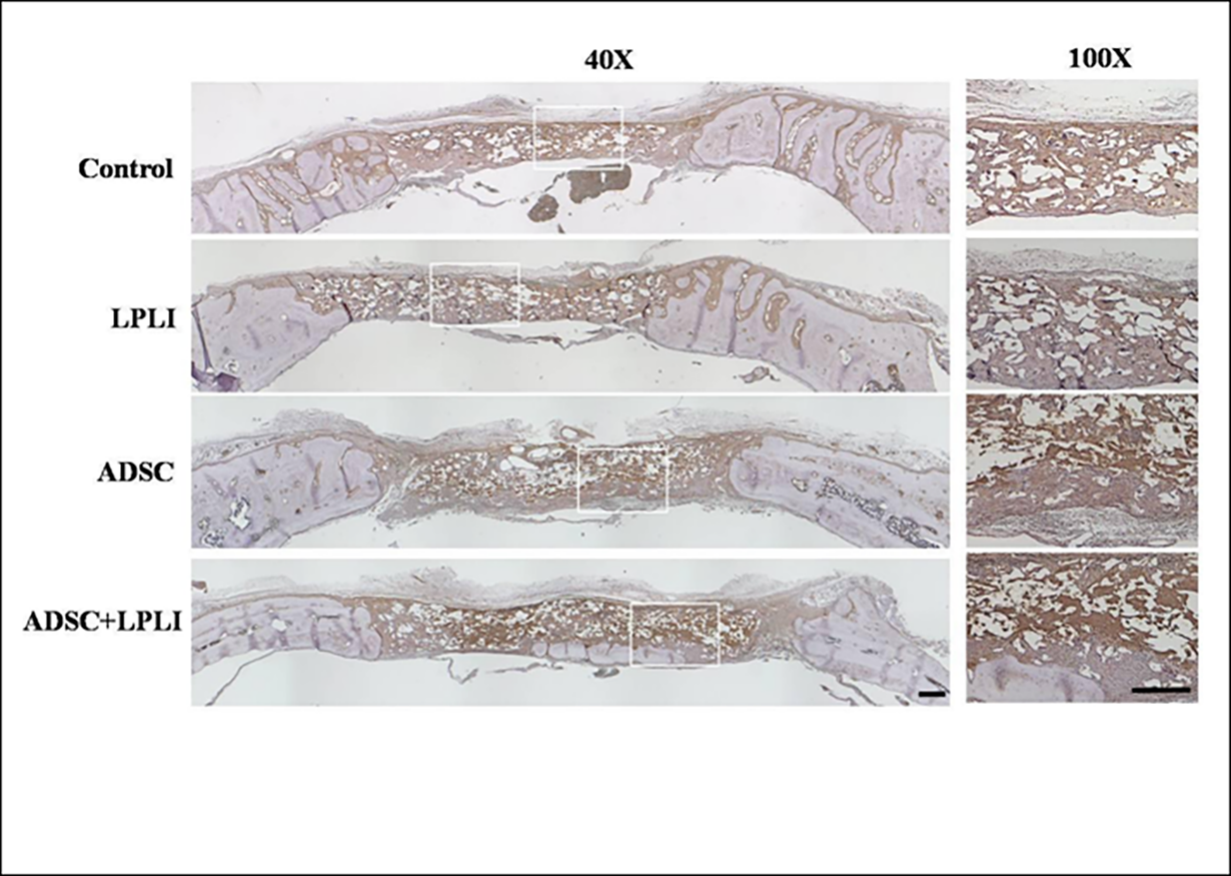
IHC staining of osteogenic factor osteocalcin of the calvarial defects at 16 weeks post-operation. The calvarial bone sections were stained for osteocalcin. Right panel, representative sections with a higher magnification in each group. *Bar*, 500 μm.

Immunohistomorphology was further applied to detect the expression levels of blood vessel marker vWF (Fig 5) and osteogenic factors, BMP-2 (Fig 6), collagen I (Fig 7) and osteocalcin (Fig 8) at 16 weeks post-operation. The control group did not show the signal of the vWF expression, but weak signals of vWF were detected in the LPLI group. Interestingly, the ADSC and ADSC+LPLI groups showed strong signals of vWF expression at week 16 (Fig 5). In contrast, the signals of the osteogenic factors were detected in all groups, and all the osteogenic factors showed no obvious difference among these groups (Figs 6–8).

## Discussion

Our present findings highlight the effects of the ADSC and/or LPLI treatments on stimulating bone tissue regeneration and inducing bone fracture healing. A number of studies have reported that LPLI contributes to enhancing bone formation through stimulating the proliferation of osteogenic cells, modulating the function of osteocytes and stimulating the differentiation of committed precursor cells, leading to an increase in the differentiated osteoblastic cell numbers and osteoid tissue [15, 19, 37, 38]. LPLI therapy also enhances alkaline phosphatase activity and improves the gene expression of osteocalcin [29]. Correspondingly, our results have demonstrated that the LPLI treatment significantly accelerates the rat calvarial bone defect healing compared to that of the non-treated control group when assessed by H&E analysis. Incremental bone healing after LPLI treatment could also be observed in the micro-CT analyses. Nevertheless, the positive effects of LPLI on bone are still under debate. Previous studies have reported conflicting results, in which delayed fracture healing or no effects were observed after LPLI treatments [29, 39]. While this effect has not been elucidated in the present study, it is interesting to further investigate whether the current observations are due to the aforementioned effects of LPLI on various genes and cells playing roles in osteogenesis.

In the comparative model, we observed that the implantation of ADSCs seeded onto a PLGA scaffold is capable of promoting the calvarial bone defect repair. The results are consistent with many preceding studies providing robust scientific evidence on the osteogenic potency of ADSCs and their ability to promote bone repair and regeneration *in vivo* [40–44]. ADSCs have great potential for bone regeneration due to their many appreciated features, including their increased accessibility and resistance to senescence and malignant transformation [45–47]. The secretome of the ADSCs contains several endocrine factors with bone remodeling activity [48–50]. Among these, vascular endothelial growth factor (VEGF), which is the key mediator of angiogenesis, greatly contributes to bone formation due to the bone vascularization required during expansion and repair [51, 52]. In the present study, the expression of the angiogenic factor vWF was monitored at week 16. We found that ADSC treatment with or without LPLI induced the strong expression of vWF. This finding indicates that ADSCs have a beneficial effect on angiogenesis.

In the present study, the expression levels of osteogenic factors, BMP-2, collagen I, and osteocalcin were investigated. We observed no visible differences among the experimental groups. As the experimental samples were assessed only at the experiment end point at week 16, the protein expression of the osteogenic factors might have been mitigated or expressed at an earlier time point during the bone formation process. More time points of the bone healing process should be analyzed to reveal the expression patterns of the osteogenic factors.

As our results demonstrated, the implanted ASDCs and LPLI work synergistically to increase bone formation compared to defect healing in the non-treated samples and samples receiving only ADSC or LPLI treatment. Previous studies have shown that LPLI enhances the survival of ADSCs during the wound healing process and stimulates the secretion of growth factors, including VEGF, which is an important factor during osteogenesis [53]. However, we did not an joined additional effect on the defective healing when combining ADSC and LPLI treatments in our rat calvarial defect model. In addition, no distinct differences between the LPLI and ADSC treatments were observed from the micro-CT analysis on bone formation; H&E analysis, however, revealed that ADSC treatment alone delivered a significantly greater defect in the healing capacity than LPLI treatment in our model. This effect could also be due to the cell amounts and dose of the treatments applied in the present study; further optimization will therefore be required to achieve the optimal effects desired. Nonetheless, the detailed mechanisms underlying this process remain undetermined. Future investigations should be performed to address the underlying mechanisms of the osteoinductive capability of the respective application of bone defect healing and determine how these therapies can work together as a feasible treatment.

In summary, we applied human ADSC and LPLI therapies for the treatment of a critical-sized calvarial defect in a rat model. Interestingly, combined treatment with ADSCs and LPLI could further enhance the bone healing process. These results confirmed that ADSCs and LPLI treatments could improve the fracture repair *in vivo*, suggesting that the combined treatment with ADSCs and LPLI is a promising strategy, especially for the treatment of compound fractures, large bone defects and non-union bone defects.

## Supporting information

S1 Fig The different diameters of the ROI scopes used in the micro-CT analysis for both parameters. The upper arrow, shows bone volume (diameter of ROI = 7mm) and the lower arrow, defect area (diameter of ROI = 9mm).
(JPG)
