# Supplementary figures and images for: Low power laser irradiation and human adipose-derived stem cell treatments promote bone regeneration in critical-sized calvarial defects in rats
Source: PLoS One. 2018 Apr 5;13(4):e0195337. doi: 10.1371/journal.pone.0195337 (PMC5886537; doi:10.1371/journal.pone.0195337)

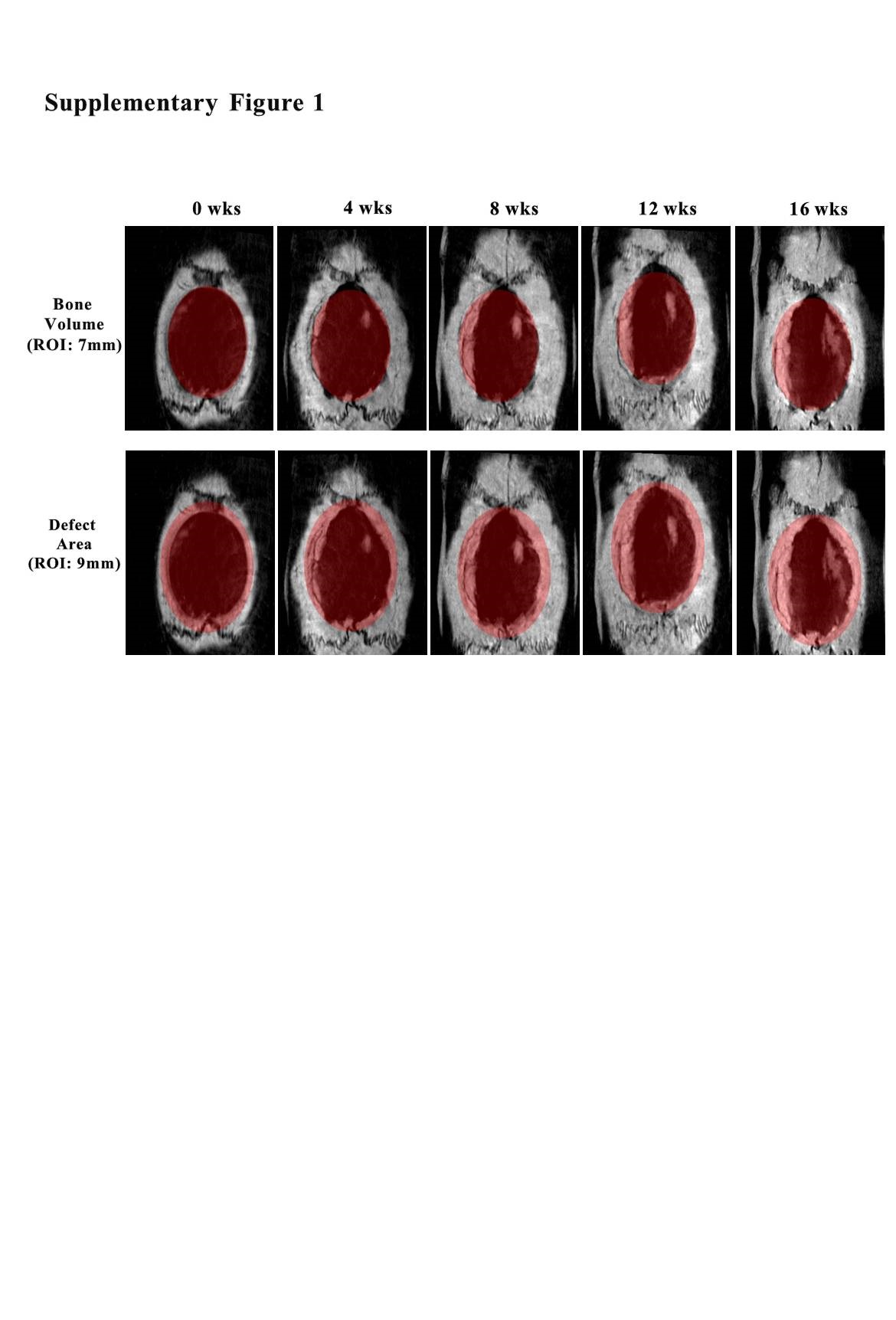

Supplement: S1 Fig — The upper arrow, shows bone volume (diameter of ROI = 7mm) and the lower arrow, defect area (diameter of ROI = 9mm). (JPG) [file pone.0195337.s001.jpg]
